# Kinetic Gas Hydrate Inhibition by Alternating Dipeptoids with Optimal Size and Shape *N*-Substituents

**DOI:** 10.1021/acsomega.4c02214

**Published:** 2024-08-09

**Authors:** Malcolm A. Kelland, Yasuhito Koyama, Janronel Pomicpic, Takuma Shinoda

**Affiliations:** † Department of Chemistry, Bioscience and Environmental Engineering, Faculty of Science and Technology, University of Stavanger, Stavanger N-4036, Norway; ‡ Department of Pharmaceutical Engineering, Faculty of Engineering, Toyama Prefectural University, 5180 Kurokawa, Imizu, Toyama 939-0398, Japan

## Abstract

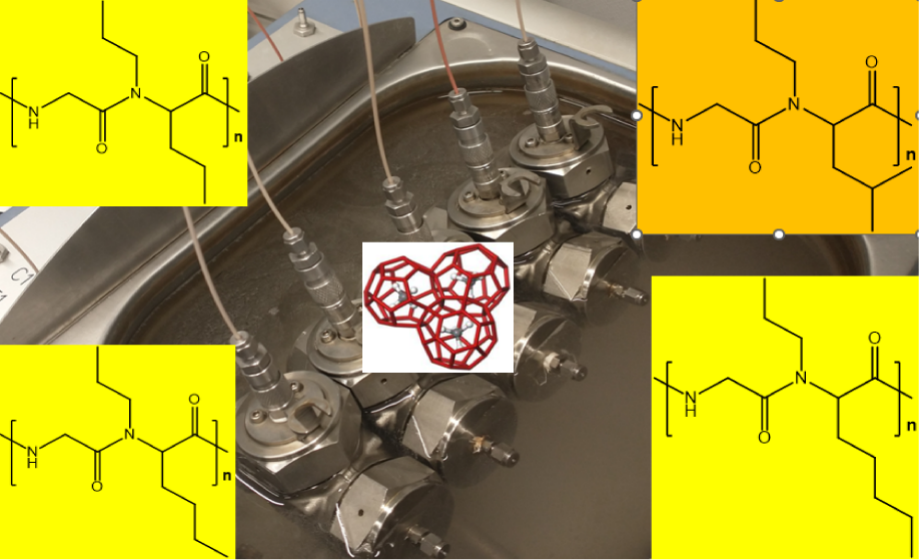

Current commercial kinetic hydrate inhibitors (KHIs) are all based on water-soluble polymers with amphiphilic alkylamide or lactam groups. The size and shape of the hydrophobic moiety are known to be critical for optimum KHI performance. Proteins and peptides represent an environmentally friendly alternative, especially as bioengineering could be used to manufacture a product predetermined to have optimum KHI performance. Here, we explore a new series of polymers that are alternating dipeptoids where one of the peptide links originates from glycine. The dipeptoids contain *n*-propyl groups on the nitrogen atom and varying size and shape alkyl side chains on the neighboring carbon atom. Experiments were carried out in high-pressure steel rocking cells using the slow constant cooling (SCC) test method (1 °C/h) and a synthetic natural gas mixture. All the dipeptoids showed good KHI performance with the best result being for that with a glycine-*N*-propylleucine repeating unit (**Poly iC4-Pr**), which has pendant iso-butyl groups on the carbon atom. It exhibited the same KHI performance as poly(*N*-vinyl caprolactam). Dipeptoids with smaller or longer alkyl groups than iso-butyl gave worse performance. It is conjectured that the iso-butyl group is the optimal carbon length for this polymer class. In addition, the end-branching maximizes the van der Waals interaction with open cavities on growing hydrate particles, which must occur without loss of hydrogen-bonding from the neighboring peptide linkage for optimum KHI performance. Thus, the study provides further evidence for the premise that good KHI molecules must contain multiple amphiphilic groups (often as polymers) with optimal size and shape hydrophobic groups adjacent to strong hydrogen bonding groups. The solvent, *n*-butyl glycol ether, was shown to be a synergist for **Poly iC4-Pr**, lowering the onset temperature of hydrate formation in SSC tests relative to the polymer alone.

## 1 Introduction

**Figure 1:**
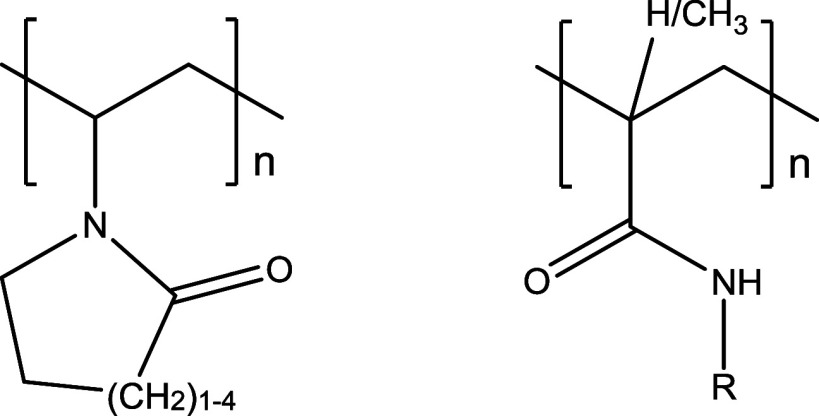
Poly(*N*-vinyl lactam)s (left) and poly(*N*-alkyl(meth)acrylamides) (right).

One of the chemical methods to prevent the formation of gas hydrate deposits and plugs in subsea flow lines is the use of kinetic hydrate inhibitors (KHIs).^1−3^ KHIs are a class of low dosage hydrate inhibitors (LDHIs) and can have several advantages over the use of thermodynamic inhibitors (THIs).^4,5^ The main advantage is the much lower concentrations required compared with THIs, resulting in much smaller storage and injection facilities. Although KHIs are not currently recycled, methods to do this have been developed. KHI formulations contain one or more water-soluble polymers, often with added synergists to boost performance. These synergists can include the KHI solvent or solvents. By definition, KHIs are a time-dependent treatment, usually limited in their application range to a subcooling of about 10–12 °C depending on a host of different field conditions. The subcooling is the difference between the actual system temperature and the equilibrium temperature at a given pressure.^1,2^ The subcooling is a measure of the driving force for hydrate formation. Increasing the subcooling at which a KHI can be deployed in the field (which is a measure of the KHI performance) is one of the goals of our research. Currently, the water-soluble polymers deployed in commercial KHI formulations are polyamides, based on *N*-vinyl lactams, *N*-alkyl(meth)acrylamides, and hyperbranched polyester amides (Figure 1). The 7-ring polymer poly(*N*-vinyl caprolactam) (PVCap) is often used as a standard by which to compare the performance of other KHI polymers.^6−16^ Only a few commercial KHI polymers are widely available for academic research purposes, and PVCap is one of them. Due to its high KHI performance, it makes a useful gauge when attempting to find novel KHI polymers with even better performance.

These polymers contain amphiphilic functional groups, which are believed to inhibit gas hydrate nucleation and crystal growth by interfering with the growth of subcritical nuclei or thermodynamically stable hydrate crystals.^7,17^ This occurs *via* hydrogen-bonding of the amides and van der Waals interactions of the hydrophobic moieties with open cavities on the hydrate particle surfaces.^18,19^ Computer modeling studies have also shown that this occurs.^20−24^ Absorption of KHI polymer onto the methane bubble surface to mitigate methane dissolution in the aqueous phase has also been proposed as a gas hydrate nucleation inhibition mechanism.^25^ Although KHIs are not used to melt hydrate plugs, there is evidence that some KHI polymers can lead to complete hydrate dissociation a few degrees Celsius into the thermodynamically stable pressure–temperature hydrate region.^26^

**Figure 2:**
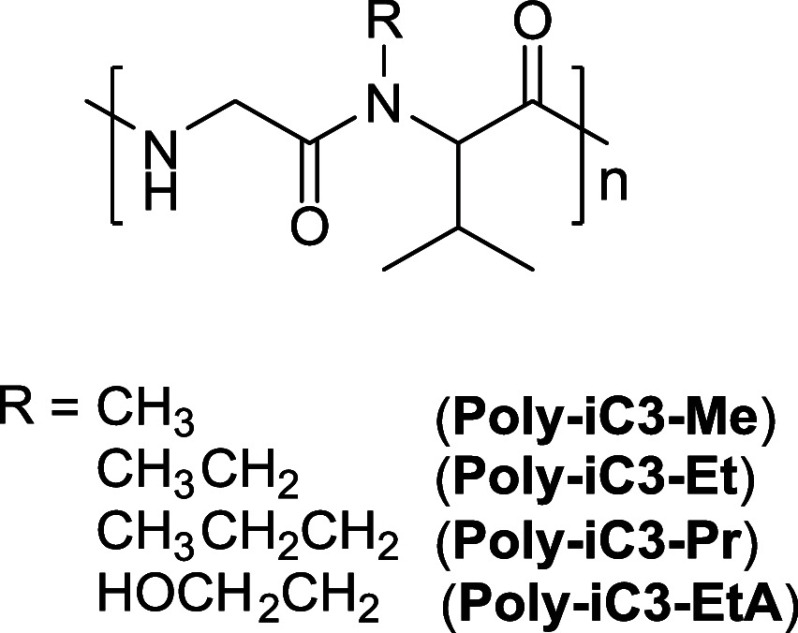
Alternating glycyl-valine-based peptoids from the previous study.^35^.

Other classes of polyamides include natural proteins and peptides, as well as synthetic varieties. These amino acid–based polymers can be thermoresponsive, a property useful for KHIs as well as other applications such as in drug delivery.^17,27^ Some of these polymers have been investigated as KHIs. These include antifreeze proteins (AFPs) and antifreeze glycoproteins (AFGPs), as well as some synthesized polypeptides and pseudopeptides.^28,29^ The polypeptides investigated were made using asparagine and the comonomers valine and leucine and gave relatively weak KHI performance.^30^ In contrast to most commercial KHI polymers, the peptide amide linkages are contained in the backbone. This reduces the molecular weight of a monomer unit, which gives a higher molar concentration of monomer units for a given weight percent polymer concentration in solution. Second, natural peptides are biodegradable, whereas the majority of commercial KHIs (mostly with a polyvinyl backbone) are poorly biodegraded. Third, none of these natural peptide molecules were designed to inhibit gas hydrate formation. This gives an opportunity to design a peptide KHI with optimal hydrophobic groups attached to the peptide backbone. The KHI peptide could then be produced by genetic engineering. This technology is already used on a large scale to produce an ice structuring protein (ISP) for use in making smoother ice cream.^31^ Recently, we reported the synthesis of a new class of pseudopolypeptides alternating glycine and substituted valine units. They were synthesized as sequenced dipeptide segments *via* our invented three-component polymerization technique using alternating peptoid precursors (Figure 2). One of the great advantages of our development is that the alternating peptide structure can be flexibly modified by changing the precursors, so it is not restricted to 20 types of natural amino acid skeletons.^32−34^ They were tested for KHI performance in high-pressure rocking cells and a structure II-forming natural gas mixture.^35^ It was shown that the pseudopeptides gave increasing performance (*i.e.*, preventing hydrate formation to increasing subcoolings) as the alkyl group on the *N*-atom was increased from methyl to ethyl to propyl but lost performance when a hydroxyethyl group was used.

Based on our initial findings, we have now expanded the range of dipeptoids in an attempt to improve KHI performance. We have synthesized dipeptoids with C3–5 groups (*n*-propyl, *n*-butyl, iso-butyl, and *n*-pentyl) as past studies have indicated that these larger hydrophobic groups could be more optimal for use in KHI polymers to improve performance. The dipeptoids exhibited both upper and lower critical solution temperatures (UCST and LCST, respectively). The critical temperature is defined as the upper critical solution temperature (UCST) when phase separation from the solution occurs at temperatures below the critical temperature, whereas it is called the lower critical solution temperature (LCST) when the phase separation occurs at temperatures above the critical temperature. We show that the best performance was found for a dipeptoid with iso-butyl groups and that the performance could be increased further by the addition of a synergist solvent.

## 2 Polymer Synthesis and Experimental Methods

Pure poly(*N*-vinylcaprolactam) (PVCap, *M*_w_ = 2000–4000 g/mol) was made from Luvicap EG, supplied by BASF, Germany, by removing the monoethylene glycol solvent. Poly(*N*-vinylpyrrolidone) (PVP, *M*_w_ = 6000–15 000 g/mol) was supplied by Ashland Chemical Company. Propylammonium chloride (Fujifilm Wako Pure Chemical Corporation, Osaka, Japan), superdehydrated isopropyl alcohol (*i*-PrOH, Fujifilm Wako Pure Chemical Corporation, Osaka, Japan), butanal (Tokyo Chemical Industry Co., Inc., Tokyo, Japan), isovaleraldehyde (Tokyo Chemical Industry Co., Inc., Tokyo, Japan), pentanal (Tokyo Chemical Industry Co., Inc., Tokyo, Japan), and hexanal (Tokyo Chemical Industry Co., Inc., Tokyo, Japan) were used as obtained. Molecular sieves 3 Å (MS 3A, Fujifilm Wako Pure Chemical Corporation, Osaka, Japan) as dehydrator were activated by careful heating using a heat gun for 5 min under vacuum. Potassium isocyanoacetate was synthesized according to the literature.^36^

### 2.1 Polymer Synthesis

**Figure 3:**
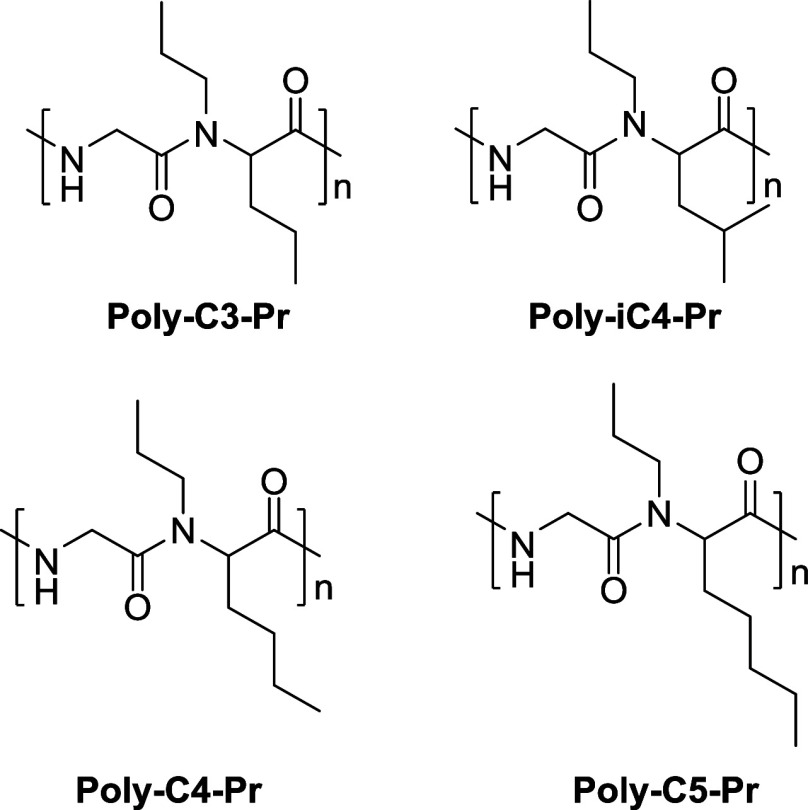
New alternating dipeptoids were made for this study.

The structures of all newly synthesized polymers are given in Figure 3.

Synthesis of an alternating peptoid with glycine-*N*-propylpropylglycine as a repeating unit (**Poly-C3-Pr**): propylammonium chloride (1.43 g, 15.0 mmol), potassium isocyanoacetate (1.85 g, 15.0 mmol), and activated MS 3A (500 mg) were added to dried *i*-PrOH (7.5 mL). After stirring for 1 h at room temperature, butanal (1.34 mL, 15.0 mmol) was added to the mixture. The mixture was stirred for 3 daysat room temperature, heated to 60 °C, and further stirred for 2 d. The reaction mixture was cooled to room temperature, diluted with THF, and filtered through a Celite pad. The filtrate was concentrated *in vacuo* to give the crude material. The crude was dissolved with a small amount of CHCl_3_, and the solution was precipitated into hexane and decanted. The residue was dried *in vacuo* to give the hexane-insoluble part (**Poly-C3-Pr**, 2.15 g, 72%) as an amorphous solid: *M*_n_ 2500 g/mol (estimated by ^1^H NMR spectroscopy); ^1^H NMR (400 MHz, CF_3_COOD, 298 K) δ 4.28–3.09 (m, 5H, *N*-CH, *N*-CH_2_), 2.16–0.38 (m, 12H, CH_2_, CH_3_) ppm; ^13^C NMR (100 MHz, CDCl_3_, 298 K, amide *S-trans* and *S-cis* rotamers) δ 168.3, 165.1, 60.6, 60.4, 48.0, 41.6, 41.4, 32.0, 29.7, 22.65, 22.57, 21.7, 20.0, 18.5, 13.9, 13.8, 11.4, 11.1 ppm; IR (ATR) υ 3272 (NH), 1652 (C=O) cm^–1^.

Synthesis of an alternating peptoid with glycine-*N*-propylleucine as a repeating unit (**Poly iC4-Pr**): propylammonium chloride (1.43 g, 15.0 mmol), potassium isocyanoacetate (1.85 g, 15.0 mmol), and activated MS 3A (500 mg) were added to dried *i*-PrOH (7.5 mL). After the mixture was stirred for 1 h at room temperature, isovaleraldehyde (1.61 mL, 15.0 mmol) was added to the mixture. The mixture was stirred for 3 days at room temperature, heated to 60 °C, and further stirred for 2 d. The reaction mixture was cooled to room temperature, diluted with THF, and filtered through a Celite pad. The filtrate was concentrated *in vacuo* to give the crude material. The crude was dissolved with a small amount of CHCl_3_ and the solution was precipitated into hexane and decanted. The residue was dried *in vacuo* to give the hexane-insoluble part (**Poly iC4-Pr**, 1.90 g, 60%) as an amorphous solid: *M*_n_ 3000 g/mol (estimated by ^1^H NMR spectroscopy); ^1^H NMR (400 MHz, CF_3_COOD, 298 K) δ 4.29–3.08 (m, 5H, *N*-CH, *N*-CH_2_), 2.16–0.13 (m, 14H, CH_2_, CH_3_) ppm; ^13^C NMR (100 MHz, CDCl_3_, 298 K, amide *S-trans* and *S-cis* rotamers) δ 168.3, 167.4, 165.0, 59.6, 59.3, 47.5, 41.6, 41.4, 36.7, 27.9, 24.9, 24.7, 23.4, 22.7, 22.5, 21.7, 20.3, 11.5, 11.2 ppm; IR (ATR) υ 3269 (NH), 1652 (C=O) cm^–1^.

Synthesis of an alternating peptoid with glycine-*N*-propylbutylglycine as a repeating unit (**Poly-C4-Pr**): propylammonium chloride (1.43 g, 15.0 mmol), potassium isocyanoacetate (1.85 g, 15.0 mmol), and activated MS 3A (500 mg) were added to dried *i*-PrOH (7.5 mL). After the mixture was stirred for 1 h at room temperature, pentanal (1.58 mL, 15.0 mmol) was added to the mixture. The mixture was stirred for 3 d at room temperature, heated to 60 °C, and further stirred for 2 d. The reaction mixture was cooled to room temperature, diluted with THF, and filtered through a Celite pad. The filtrate was concentrated *in vacuo* to give the crude material. The crude was dissolved with a small amount of CHCl_3_, and the solution was precipitated into hexane and decanted. The residue was dried *in vacuo* to give the hexane-insoluble part (**Poly-C4-Pr**, 2.40 g, 75%) as an amorphous solid: *M*_n_ 2700 g/mol (estimated by ^1^H NMR spectroscopy); ^1^H NMR (400 MHz, CF_3_COOD, 298 K) δ 4.29–3.07 (m, 5H, *N*-CH, *N*-CH_2_), 2.21–0.15 (m, 14H, CH_2_, CH_3_) ppm; ^13^C NMR (100 MHz, CDCl_3_, 298 K, amide *S-trans* and *S-cis* rotamers) δ 168.7, 168.3, 165.0, 65.8, 60.5, 48.2, 47.9, 41.6, 41.4, 30.5, 27.6, 27.2, 22.7, 22.6, 22.51, 22.46, 20.1, 15.2, 13.8, 13.7, 11.4, 11.1 ppm; IR (ATR) υ 3279 (NH), 1652 (C=O) cm^–1^.

Synthesis of an alternating peptoid with glycine-*N*-propylpentylglycine as a repeating unit (**Poly-C5-Pr**): propylammonium chloride (1.43 g, 15.0 mmol), potassium isocyanoacetate (1.85 g, 15.0 mmol), and activated MS 3A (500 mg) were added to dried *i*-PrOH (7.5 mL). After the mixture was stirred for 1 h at room temperature, hexanal (1.83 mL, 15.0 mmol) was added to the mixture. The mixture was stirred for 3 d at room temperature, heated to 60 °C, and further stirred for 2 d. The reaction mixture was cooled to room temperature, diluted with THF, and filtered through a Celite pad. The filtrate was concentrated *in vacuo* to give the crude material. The crude was dissolved with a small amount of CHCl_3_, and the solution was precipitated into hexane and decanted. The residue was dried *in vacuo* to give the hexane-insoluble part (**Poly-C5-Pr**, 2.05 g, 60%) as an amorphous solid: *M*_n_ 2800 g/mol (estimated by ^1^H NMR spectroscopy); ^1^H NMR (400 MHz, CF_3_COOD, 298 K) δ 4.29–3.10 (m, 5H, *N*-CH, *N*-CH_2_), 2.19–0.13 (m, 16H, CH_2_, CH_3_) ppm; ^13^C NMR (100 MHz, CDCl_3_, 298 K, amide *S-trans* and *S-cis* rotamers) δ 169.1, 168.6, 165.0, 60.8, 60.9, 48.3, 43.3, 41.6, 41.2, 31.6, 31.5, 28.1, 27.8, 24.9, 24.7, 22.6, 22.4, 20.2, 19.9, 13.91, 13.87, 11.4, 11.2 ppm; IR (ATR) υ 3282 (NH), 1652 (C=O) cm^–1^ (Table 1).

**Table 1:** New Alternating Dipeptoids Synthesized for This Study.^a^

polymer	*M*_n_ (g/mol)	LCST or UCST upon heating	LCST upon cooling
**Poly-C3-Pr**	2500	UCST = 60 °C	LCST = 20 °C
**Poly iC4-Pr**	3000	UCST = 50 °C	LCST = 10 °C
**Poly-C4-Pr**	2700	UCST = 50 °C	LCST = 10 °C
**Poly-C5-Pr**	2800	LCST = 30 °C	LCST = 20 °C

a The polymer sample (10.0 mg) was first dissolved in EtOH (100 μL) and then diluted with H_2_O (1.9 mL). The UV–vis spectra of the sample aqueous solutions were collected at 10 °C intervals upon heating from 30 to 90 °C and upon cooling from 70 to 10 °C. The waiting time for each temperature was 5 min. LCST, lower critical solution temperature; UCST:, upper critical solution temperature.

### 2.2 Spectral Measurements

The ^1^H NMR (400 MHz) and ^13^C NMR (100 MHz) spectra were recorded on a JEOL ECZ400R spectrometer (JEOL Co. Ltd., Tokyo, Japan) using CD_3_COOD or CDCl_3_ as a solvent and calibrated using residual undeuterated solvent as an internal standard. FT-IR spectra *via* an attenuated total reflection (ATR) method were measured using an IR Spirit spectrometer (Shimadzu Co. Ltd., Kyoto, Japan). Turbidity measurements were performed with a JASCO V-750ST spectrophotometer (JASCO Co. Ltd., Tokyo, Japan) using a 0.1 cm path quartz cell with a temperature controller (ETCS-761, JASCO Co. Ltd., Tokyo, Japan).

### 2.3 KHI Experimental Test Procedure

**Figure 4:**
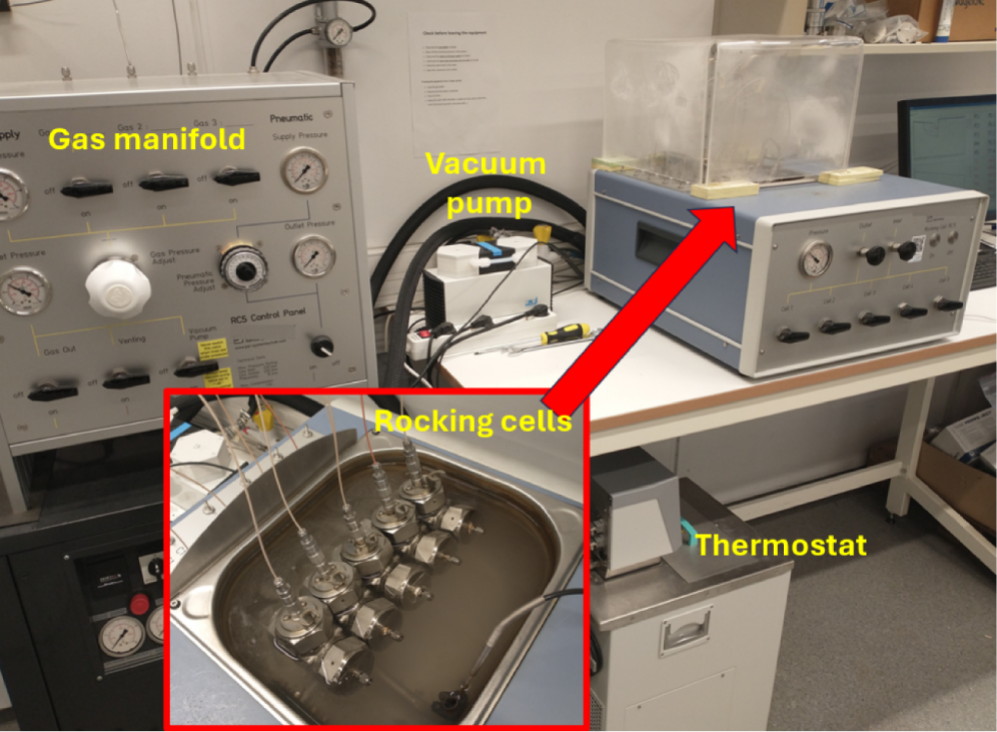
High pressure KHI test equipment. The inset shows the five rocking cells in the water bath.

All KHI tests to determine the relative performance ranking of the new polymers were carried out in five parallel 40 mL stainless steel cells. The cells are placed in a temperature-controlled water bath. (Figure 4).^17^ The whole equipment was supplied by PSL Systemtechnik, Germany, except the steel cells, which were manufactured by Swafas. Each steel cell contains a steel ball that provides agitation to the test solution when the cell is rocked. Each cell also has its own pressure and temperature sensor, as well as a temperature sensor in the water bath. The cells were pressurized with synthetic natural gas (SNG, Table 2). This gas preferentially forms structure II gas hydrates as the most thermodynamically stable phase.^1,2^

**Table 2:** Synthetic Natural Gas (SNG) Composition

component	mol %
nitrogen	0.11
*n*-butane	0.72
isobutane	1.65
propane	5.00
CO_2_	1.82
ethane	10.3
methane	80.4

Using the rocking cells, aqueous solutions of polymers were evaluated for their KHI performance by the slow constant cooling (SCC) experimental method. This is summarized below^:^

1. A polymer was dissolved to the desired concentration, usually 2500 ppm, in deionized water until fully dissolved.

2. 20 mL aliquot of an aqueous polymer solution was added to each of the five cells.

3. Using repeated vacuum and pressurizing with SNG, the air in the cells was replaced with SNG up to 76 bar.

4. The cells were rocked at a rate of 20 rocks per minute with an angle of 40°, while being cooled at 1.0 °C/h from 20.5 to 2.0 °C.

**Figure 5:**
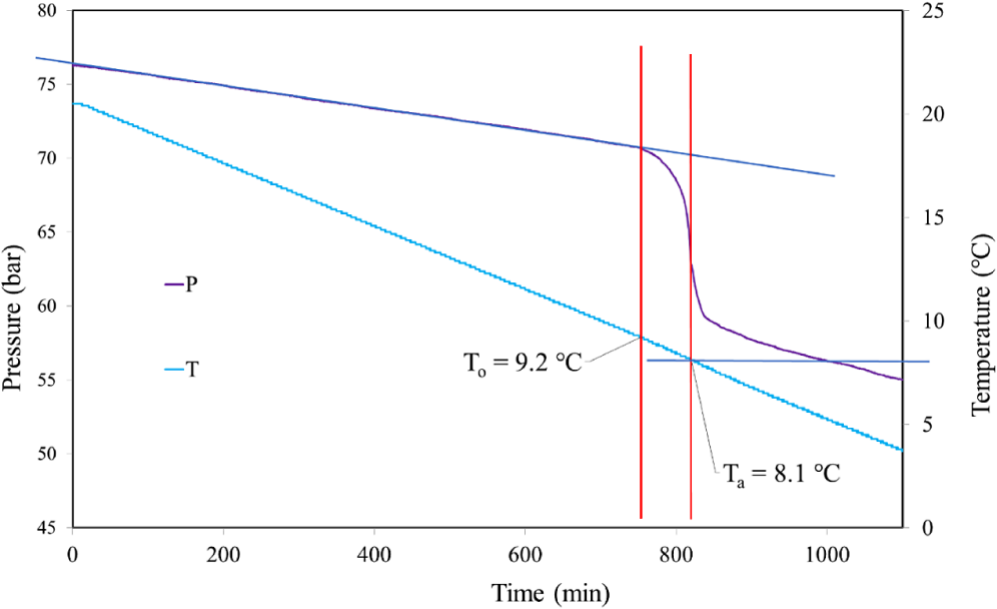
Determination of *T*_o_ and *T*_a_ values for one rocking cell constant cooling experiment with 2500 ppm of **Poly iC4-Pr** and 10 000 ppm of BGE.

From previous studies, the hydrate equilibrium temperature (*T*_eq_) at 76 bar was found to be 20.2 ± 0.05 °C, determined by slow dissociation experiments warming at 0.025 °C/h for the last 3–4 °C.^37^ This value correlates well with calculations carried out with PVTSim software (Calsep, Denmark). During the constant cooling period, a linear pressure decrease occurs until the first detected onset of hydrate formation (*T*_o_). At this temperature, the pressure drops faster due to the SNG being used to make gas hydrates (Figure 5). The start of nucleation may possibly happen earlier than *T*_o_ but is not accurately detectable. *T*_a_ is taken as the temperature when the pressure decrease due to hydrate formation is most rapid. In the experimental result in Figure 4 (for 2500 ppm of **Poly iC4-Pr** and 10 000 ppm of BGE), *T*_o_ was determined as 9.2 °C and *T*_a_ as 8.2 °C. The standard deviation (assuming a normal distribution) for a set of 5–10 *T*_o_ or *T*_a_ values is no more than 0.6 °C and usually less than 0.3 °C. The scattering still allows for a rough ranking of the performance of the KHI samples as long as sufficient tests are carried out for a statistically significant difference using a *t* test. Depending on the variation in average *T*_o_ between samples, 5–10 tests, as per this study, is usually sufficient to get a significant difference at the 95% confidence level (*p* < 0.05).^38^

## 3 Results and Discussion

A set of alternating dipeptoids with tailor-made *N*-substituents was synthesized (Figure 3). These polymers showed lower or upper critical solution temperature (LCST or UCST) properties in solution. The results are summarized in Table 1. Low concentrations of polymer, as in the KHI experiments at 2500 ppm, would be soluble in an aqueous solution even below UCST temperature. The equilibrium is determined by the critical aggregation concentration (CAC). Therefore, at around CAC, all of the polymer can dissolve in water or form small associates such as micelles.

The KHI test results are summarized in Table 3. Results for deionized water, PVP, PVCap, and three related previously tested dipeptoids are added to the table for comparison. Operators of flowlines that are prone to gas hydrate formation want to completely stop any macroscopic buildup of hydrates. Therefore, the *T*_o_ value is chosen as the main parameter to evaluate and compare the performance of KHIs, *i.e.*, the lower the *T*_o_ value (or higher subcooling), the better the KHI performance. The *T*_o_ – *T*_a_ value can give some indication of the ability of a KHI to slow the crystal growth process, but KHIs can be compared only if the driving forces when macroscopic growth starts are roughly equal. It is clear from the average *T*_o_ values that all polymers tested gave significantly better performance than no additive. PVCap performs better than PVP due to the larger hydrophobic ring which is also better optimized for interaction with hydrate particle surfaces. The previously tested dipeptoids **Poly iC3-Me**, **Poly iC3-Et**, and **Poly iC3-Pr** all contain carbon-bonded iso-propyl groups as side chains in addition to methyl, ethyl, and propyl groups on the nitrogen atom, respectively. These polymers showed increasing performance as the size of the hydrophobic alkyl side chains was increased. Increasing the size of the hydrophobic side chains also lowers the LCST which has been shown to give increased KHI performance as long as the alkyl groups are of optimum size and shape.

**Table 3:** Results of Slow Constant Cooling KHI Experiments for Polymers at 2500 ppm

additive	concn. (ppm)	*T*_o_ (av.) (°C)	*T*_a_ (av.) (°C)	*T*_o_ – *T*_a_ (av.) (°C)
deionized water		16.9	16.8	0.1
PVP	2500	13.9	10.3	3.6
PVCap	2500	10.4	9.9	0.5
**Poly iC3-Me**(17,20)	2500	13.0	12.9	0.1
**Poly iC3-Et**(19,20)	2500	13.3	13.0	0.3
**Poly iC3-Pr**(19,20)	2500	10.4	10.2	0.2
**Poly-C3-Pr**	2500	13.2	11.9	1.3
**Poly-C4-Pr**	2500	13.2	12.3	0.9
**Poly-C5-Pr**	2500	14.6	12.9	1.7
**Poly iC4-Pr**	1000	12.3	11.7	0.6
**Poly iC4-Pr**	2500	10.4	9.3	1.1
**Poly iC4-Pr**	2500 + 10 000 BGE	9.1	8.1	1.0

The four new dipeptoids are variations of **Poly iC3-Pr** in that they have the same nitrogen-bonded *n*-propyl group but have other sizes of alkyl groups bonded to a carbon atom on the backbone. The *n*-propyl group in all the dipeptoids adds hydrophobic character in addition to a second alkyl group with 3–5 carbon atoms, which gives all the new polymers low LCST values.

The best performance among the new dipeptoids was for **Poly iC4-Pr** giving an average *T*_o_ value of 10.4 °C, the same value as that obtained for **Poly iC3-Pr**. Both dipeptoids contain branched alkyl groups, iso-butyl and iso-propyl, respectively. End-branching, of the correct size, improves the van der Waals interaction of the alkyl group with open hydrate cavities on the growing hydrate particle surfaces, enhancing the KHI effect. Iso-propyl has been shown to be an excellent alkyl group in KHI polymers such as *N*-isopropyl(meth)acrylamides, and the iso-butyl group has been used successfully in maleamide polymers to similar effect.^39^ The worst performance of the four new dipeptoids was for **Poly-C5-Pr**. Although it has a low LCST value, which can be useful for the KHI performance, we believe the *n*-pentyl group in this polymer is too long for optimum interaction with gas hydrate surfaces.^17^ The neighboring amide group cannot hydrogen-bond to the gas hydrate surface as easily and strongly as for dipeptoids with side groups of 3–4 carbon atom chain lengths (*i.e.*, the other three new dipeptoids). Decreasing the alkyl group further by 1–2 carbon atoms (methyl or ethyl) as seen for **Poly iC3-Me** or **Poly iC3-Et** would decrease the KHI performance because these short alkyl chains cannot penetrate as well into hydrate cavities as the alkyl groups of 3–4 carbon atom length (causing weaker van der Waals interactions). Therefore, this study shows there is an optimal chain length for the best KHI performance, and that end-branching of the alkyl group improves the performance further.^17^ The difference between the *T*_o_ and *T*_a_ values, which gives a rough measure of the ability to arrest hydrate crystal growth, is in the range of typical values seen with other KHI polymer classes.

In general, KHIs perform better with increasing concentration, certainly within the 1000–5000 ppm range. This was also true of **Poly iC4-Pr**, which gave an average *T*_o_ value 1.9 °C lower at 1000 ppm compared to the result at 2500 ppm. We also wanted to determine whether the KHI performance of these dipeptoids could be improved by the solvent. Several studies have shown that *n*-butyl glycol ether (BGE) acts as a solvent synergist for both substituted polyacrylamides and poly(*N*-vinyl lactams).^40−42^ A synergist usually has no significant KHI effect by itself, but its addition to a KHI polymer improves the performance. For the slow constant cooling test method, this means the average *T*_o_ value will be significantly lowered by adding the synergist. Indeed, some commercial KHI polymers are sold in this high flash point mutual solvent.^43,44^ A solution of 2500 ppm of **Poly iC4-Pr** with added 10 000 ppm of BGE was made. This would mean the concentrated blend would be 80% solvent and 20% polymer, which is a typical concentration with a low enough viscosity for injection and pumping purposes. The addition of BGE lowered the average *T*_o_ value by 1.3 °C compared to testing the dipeptoid **Poly iC4-Pr** at 2500 ppm by itself. The difference was statistically significant at the 95% certainty level from a statistical *t* test using 5 tests with BGE and 5 tests without BGE. A small thermodynamic effect from the BGE must be taken into account, but this is not enough by itself to cause the KHI performance improvement. BGE has been known to lower the average *T*_o_ value in identical test equipment and test methods by up to 3 °C when added at 10 000 ppm BGE to 2500 ppm PVCap.^45^

The mechanism of synergy between KHI polymers and small molecules such as BGE is unclear. The size and shape of the alkyl group in the synergist appear to be critical, just as the size of the hydrophobic groups in KHI polymers also need to be of optimum size and shape.^17,40^ Suggestions have been made that the glycol ether synergists such as BGE function by either enhancing the absorption of the KHI polymer on hydrate growth sites more significantly than on hydrate nucleation sites, or that they stabilize the KHI polymer at the hydrate–water interface.^46,47^ The coiling of the KHI polymer may also be affected by the synergist which in turn could change the surface area-to-weight ratio enabling increased interactions with gas hydrate particle surfaces.^43^ The alkyl groups of the synergistic molecules such as BGE or leucine may also be interfering with the dissolution of gas bubbles in the aqueous phase, enhancing nucleation inhibition.^25,48^

## 4 Conclusion

A series of alternating dipeptoids with tailor-made *N*-substituents has been synthesized and characterized. They exhibited either LCST or UCST properties in an aqueous solution. All of the new dipeptoids were tested for KHI performance in high-pressure steel rocking cells using a natural gas mixture and the slow constant cooling test method. All new dipeptoids showed good KHI performance with the best result being for **Poly iC4-Pr**. This dipeptoid has the optimal size and end-branched shape iso-butyl group, maximizing the van der Waals interaction with open cavities on growing hydrate particles without losing good hydrogen-bonding from the neighboring peptide linkage. **Poly iC4-Pr** performed better at 2500 ppm compared to 1000 ppm, and BGE gave a synergistic improvement with 1000 ppm BGE, lowering the average *T*_o_ value to 9.1 °C. This is 7.8 °C better than no additive and approximately 11.2 °C lower than the equilibrium temperature. The study shows that thermoresponsive amino acid–based polymers are effective KHIs and that the route used here allows for tailored optimization.

## Supporting Information Available

^1^H NMR, ^13^C NMR, IR, and UV–vis spectra of new polymers (PDF)

## References

[ref1] Tohidi B. Gas Hydrates and Flow Assurance; Advances in Chemical and Process Engineering; World Scientific Publishing: London, UK, 2022.

[ref2] Sloan E.D.; Koh C. A. Clathrate Hydrates of Natural Gases, 3rd ed.; CRC Press: Boca Raton, FL, 2008.

[ref3] Kondapi P.; Moe R. Today’s Top 30 Flow Assurance Technologies: Where Do They Stand?; Offshore Technology Conference, 2013.

[ref4] Asheesh K. Perspectives of Flow Assurance Problems in Oil and Gas Production: A Mini-review. Energy Fuels 2023, 37 (12), 8142–8159. 10.1021/acs.energyfuels.3c00843.

[ref5] Frenier W. F.; Ziauddin M. Chemistry for Enhancing the Production of Oil and Gas, SPE Books; Society of Petroleum Engineers, 2013.

[ref6] Kelland M. A. History of the development of low dosage hydrate inhibitors. Energy Fuels 2006, 20, 825–847. 10.1021/ef050427x.

[ref7] Kelland M. A. A review of kinetic hydrate inhibitors: Tailormade water-soluble polymers for oil and gas industry applications. Advances in Materials Science Research Wytherst M. C. ed.;Nova Science Publishers, Inc: New York; 2011.

[ref8] Perrin A.; Musa O. M.; Steed J. W. The chemistry of low dosage clathrate hydrate inhibitors. Chem. Soc. Rev. 2013, 42, 1996–2015. 10.1039/c2cs35340g. 23303391

[ref9] Ankur S.; Ajay S. Review of Kinetic Hydrate Inhibitors Based on Cyclic Amides and Effect of Various Synergists. Energy Fuels 2021, 35 (19), 15301–15338. 10.1021/acs.energyfuels.1c02180.

[ref10] Chambers L. I.; Hall A. V.; Musa O. M.; Steed J. W. Hydration Behavior of Polylactam Clathrate Hydrate Inhibitors and their Small-Molecule Model Compounds. In Handbook of Pyrrolidone and Caprolactam Based Materials; Wiley Online Library: 2021, pp.1127 1169.

[ref11] Singh A.; Suri A. A review on gas hydrates and kinetic hydrate inhibitors based on acrylamides. J. Nat. Gas Sci. Eng. 2020, 83, 103539 10.1016/j.jngse.2020.103539.

[ref12] Zhang Q.; Cai W.; Li Z.; Lu H. Insights into behaviors of guest and host molecules in methane hydrate formation process in the presence of kinetic inhibitors via in-situ micro-Raman spectroscopy. Fuel 2024, 358 (Part A), 130195 10.1016/j.fuel.2023.130195.

[ref13] Li Y.; Li Y.; Shen L.; Wang Y.; Wei N.; Shen X. Effects of PVP and PVCap in Brine Solutions on the Formation Kinetics and Morphological Evolution of Sour Gas Hydrates. Energy Fuels 2023, 37 (19), 14906–14913. 10.1021/acs.energyfuels.3c02745.

[ref14] Semenov A. P.; Gong Y.; Medvedev V. I.; Stoporev A. S.; Istomin V. A.; Vinokurov V. A.; Li T. New insights into methane hydrate inhibition with blends of vinyl lactam polymer and methanol, monoethylene glycol, or diethylene glycol as hybrid inhibitors. Chem. Eng. Sci. 2023, 268, 118387 10.1016/j.ces.2022.118387. PMC9876827 36710919

[ref15] Aminnaji M.; Anderson R.; Jarrahian K.; Tohidi B. Natural Pectin and Commercial Luvicap-Bio as Green Kinetic Hydrate Inhibitors: A Comparative Evaluation by Crystal Growth Inhibition Methods. Energy Fuels 2022, 36 (24), 14898–14906. 10.1021/acs.energyfuels.2c03348.

[ref16] Imran M.; Saleem Q.; Ajwad H. A.; Makogon T. Y.; Ali S. A.; Rushaid A.; Panda S. K.; Al-Eid M.; Alawani N. A.; Aleisa R. M.; et al. Design and development of N-vinylcaprolactam copolymers as kinetic hydrate inhibitors for sour gas environments. Fuel 2022, 311, 122497 10.1016/j.fuel.2021.122497.

[ref17] Dirdal E. G.; Kelland M. A. Does the Cloud Point Temperature of a Polymer Correlate with Its Kinetic Hydrate Inhibitor Performance?. Energy Fuels 2019, 33, 7127–7137. 10.1021/acs.energyfuels.9b01185.

[ref18] Carver T. J.; Drew M. G. B.; Rodger P. R. Molecular dynamics calculations of N-methylpyrrolidone in liquid water. Phys. Chem. Chem. Phys. 1999, 1 (8), 1807–1816. 10.1039/a809060b.

[ref19] Anderson B. J.; Tester J. W.; Borghi G. P.; Trout B. L. Properties of Inhibitors of Methane Hydrate Formation via Molecular Dynamics Simulations. J. Am. Chem. Soc. 2005, 127 (50), 17852–17862. 10.1021/ja0554965. 16351116

[ref20] Carver T. J.; Drew M. G. B.; Rodger P. M. Characterisation of the {111} growth planes of a type II gas hydrate and study of the mechanism of kinetic inhibition by poly(vinylpyrrolidone). J. Chem. Soc., Faraday Trans. 1996, 92, 5029–5033. 10.1039/ft9969205029.

[ref21] Cheng L.; Cui J.; Li Z.; Liu B.; Ban S.; Chen G. Molecular dynamics simulation of the formation of methane hydrates in the presence of KHIs. Chem. Eng. Sci. 2021, 236, 116508 10.1016/j.ces.2021.116508.

[ref22] Liu J.; Yan Y.; Feng Y.; Liu S. Molecular mechanisms of Poly(N-alkyl methacrylamides)s as Kinetic hydrate inhibitors. Chem. Eng. Sci. 2022, 258, 117775 10.1016/j.ces.2022.117775.

[ref23] Liu J.; Wang H.; Guo J.; Chen G.; JZhong J.; Yan Y.; Zhang J. Molecular insights into the kinetic hydrate inhibition performance of Poly (N-vinyl lactam) polymers. J. Nat. Gas Sci. Eng. 2020, 83, 103504 10.1016/j.jngse.2020.103504.

[ref24] Li S.; Lv R.; Yan Z.; Huang F.; Zhang X.; Chen G.-J.; Yue T. Design of Alanine-Rich Short Peptides as a Green Alternative of Gas Hydrate Inhibitors: Dual Methyl Group Docking for Efficient Adsorption on the Surface of Gas Hydrates. ACS Sust. Chem. Eng. 2020, 8 (10), 4256–4266. 10.1021/acssuschemeng.9b07701.

[ref25] Zhong J.; Wang Z.; Li L.; Guo M.; Zhang J.; Wang F.; Zhang J.; Wang Z. Resolving hydrate inhibition mechanism: Interactions between kinetic hydrate inhibitors and CH4 bubble. Chem. Eng. J. 2024, 490, 151440 10.1016/j.cej.2024.151440.

[ref26] Aminnaji M.; Anderson R.; Hase A.; Tohidi B. Can kinetic hydrate inhibitors inhibit the growth of pre-formed gas hydrates?. Gas Sci. Eng. 2023, 109, 104831 10.1016/j.jngse.2022.104831.

[ref27] Badreldin M.; Salas-Ambrosio P.; Garanger E.; Lecommandoux S.; Harrisson S.; Bonduelle C. Thermoresponsive polymers: From natural proteins to amino acid based polymer synthesis. Prog. Polym. Sci. 2023, 147, 101752 10.1016/j.progpolymsci.2023.101752.

[ref28] Edwards A. R. A molecular modeling study of the winter flounder antifreeze peptide as a potential kinetic hydrate inhibitor. Ann. N. Y. Acad. Sci. 1994, 715, 543–544. 10.1111/j.1749-6632.1994.tb38881.x.

[ref29] Reyes F. T.; Guo L.; Hedgepeth J. W.; Zhang D.; Kelland M. A. First Investigation of the Kinetic Hydrate Inhibitor Performance of Poly(*N*-alkylglycine)s. Energy Fuels 2014, 28, 6889–6896. 10.1021/ef501779p.

[ref30] Kelland M. A.; Zhang Q.; Chua P. C. A Study of Natural Proteins and Partially Hydrolyzed Derivatives as Green Kinetic Hydrate Inhibitors. Energy Fuels 2018, 32, 9349–9357. 10.1021/acs.energyfuels.8b02239.

[ref31] Meldolesi A. GM fish ice cream. Nat. Biotechnol. 2009, 27, 682 10.1038/nbt0809-682b.19668159

[ref32] Ihsan A. B.; Koyama Y. Impact of polypeptide sequence on thermal properties for diblock, random, and alternating copolymers containing a stoichiometric mixture of glycine and valine. Polymer 2019, 161, 197–204. 10.1016/j.polymer.2018.12.021.

[ref33] Koyama Y.; Ihsan A. B.; Gudeangadi P. G. Synthetic Approach of Thermally Tunable Nature-Mimetic Polypeptides from N-Protected Alternating Peptoids. Macromol. Chem. Phys. 2018, 219 (19), 1800303 10.1002/macp.201800303.

[ref34] Ihsan A. B.; Nargis M.; Koyama Y. Effects of the Hydrophilic–Lipophilic Balance of Alternating Peptides on Self-Assembly and Thermo-Responsive Behaviors. Int. J. Mol. Sci. 2019, 20, 4604 10.3390/ijms20184604. 31533361 PMC6770757

[ref35] Zhang Q.; Koyama Y.; Ihsan A. B.; Kelland M. A. Kinetic Hydrate Inhibition of Glycyl-valine-Based Alternating Peptoids with Tailor-Made N-Substituents. Energy Fuels 2020, 34, 4849–4854. 10.1021/acs.energyfuels.9b04321.

[ref36] Bonne D.; Dekhane M.; Zhu J. Ammonium chloride promoted Ugi four-component, five-center reaction of a-substituted a-isocyano acetic acid: a strong solvent effect. Org. Lett. 2004, 6, 4771–4774. 10.1021/ol0479388. 15575682

[ref37] Pomicpic J.; Ghosh R.; Kelland M. A. Non-Amide Polymers as Kinetic Hydrate Inhibitors - Maleic Acid/Alkyl Acrylate Copolymers and the Effect of pH on Performance. ACS Omega 2022, 7, 1404–1411. 10.1021/acsomega.1c06063. 35036801 PMC8757446

[ref38] Walpole R. E.; Myers R. H.; Myers S. L.; Walpole R. E.; Ye K. Probability and Statistics for Engineers and Scientists, 8th ed.; Pearson Education: Upper Saddle River, NJ, USA, 2007.

[ref39] Klug P.; Kelland M. Additives for inhibiting gas hydrate formation. JP 6,369,004 B1, 2002.

[ref40] Kelland M. A.; Dirdal E. G.; Ree L. H. S. Solvent Synergists for Improved Kinetic Hydrate Inhibitor Performance of Poly(N-vinylcaprolactam). Energy Fuels 2020, 34, 1653–1663. 10.1021/acs.energyfuels.9b03994.

[ref41] Ree L. H. S.; Kelland M. A. Investigation of Solvent Synergists for Improved Kinetic Hydrate Inhibitor Performance of Poly(N-isopropyl methacrylamide). Energy Fuels 2019, 33, 8231–8240. 10.1021/acs.energyfuels.9b01708.

[ref42] Kelland M. A. A Review of Kinetic Hydrate Inhibitors from an Environmental Perspective. Energy Fuels 2018, 32, 12001–12012. 10.1021/acs.energyfuels.8b03363.

[ref43] Cohen J. M.; Wolf P. F.; Young W. D. Enhanced hydrate inhibitors: powerful synergism with glycol ethers. Energy Fuels 1998, 12 (2), 216–218. 10.1021/ef970166u.

[ref44] Cohen J. M.; Wolf P. F.; Young W. D. U.S. Patent. JP 5,723,524 B2, 1998.

[ref45] Dirdal E. G.; Kelland M. A. Further Investigation of Solvent Synergists for Improved Performance of Poly(N-vinylcaprolactam)-Based Kinetic Hydrate Inhibitors. Energy Fuels 2021, 35, 20103–20116. 10.1021/acs.energyfuels.1c03567.

[ref46] Yang J.; Tohidi B. Characterization of inhibition mechanisms of kinetic hydrate inhibitors using ultrasonic test technique. Chem. Eng. Sci. 2011, 66, 278–283. 10.1016/j.ces.2010.10.025.

[ref47] Fu B. Frampton H.; Craddock H. A. The development of advanced kinetic hydrate inhibitors. In Chemistry in the Oil Industry VII: performance in a Challenging Environment; Royal Society of Chemistry: Cambridge, U.K; 2002, pp. 264 276.

[ref48] Zhang Q.; Tan Y.; Li Z.; Liang D.; Long Z.; Liu X.; Chen X.; Peng H.; Xiao J.; Shen Y. Insights into the synergistic effect of polylactam kinetic hydrate inhibitor and amino acid via molecular dynamics simulations. Energy 2024, 299, 131439 10.1016/j.energy.2024.131439.

