# Supplementary material for: Kinetic Gas Hydrate Inhibition by Alternating Dipeptoids with Optimal Size and Shape N-Substituents
Source: ACS Omega. 2024 Aug 9;9(33):35475–81. doi: 10.1021/acsomega.4c02214 (PMC11340003; doi:10.1021/acsomega.4c02214)
Supplement: Supplementary file 1 — ao4c02214_si_001.pdf [file ao4c02214_si_001.pdf]

Supplementary Materials for

Kinetic gas hydrate inhibition by alternating dipeptoids with optimum size and shape *N*-substituents

Malcolm A. Kelland,[1,*] Yasuhito Koyama,[2] Janronel Pomicpic,[1] Takuma Shinoda[2]

1 Department of Chemistry, Bioscience and Environmental Engineering, Faculty of Science and Technology, University of Stavanger, N-4036 Stavanger, Norway.

2 Department of Pharmaceutical Engineering, Faculty of Engineering, Toyama Prefectural University, 5180 Kurokawa, Imizu, Toyama 939-0398, Japan.

* Corresponding author: malcolm.kelland@uis.no

Contents

^{1}H NMR, ^{13}C NMR, and IR spectra of new polymers.

Temperature-dependency of UV-vis spectra of the polymer aqueous solutions

^{1}H NMR, ^{13}C NMR, and IR Spectra.

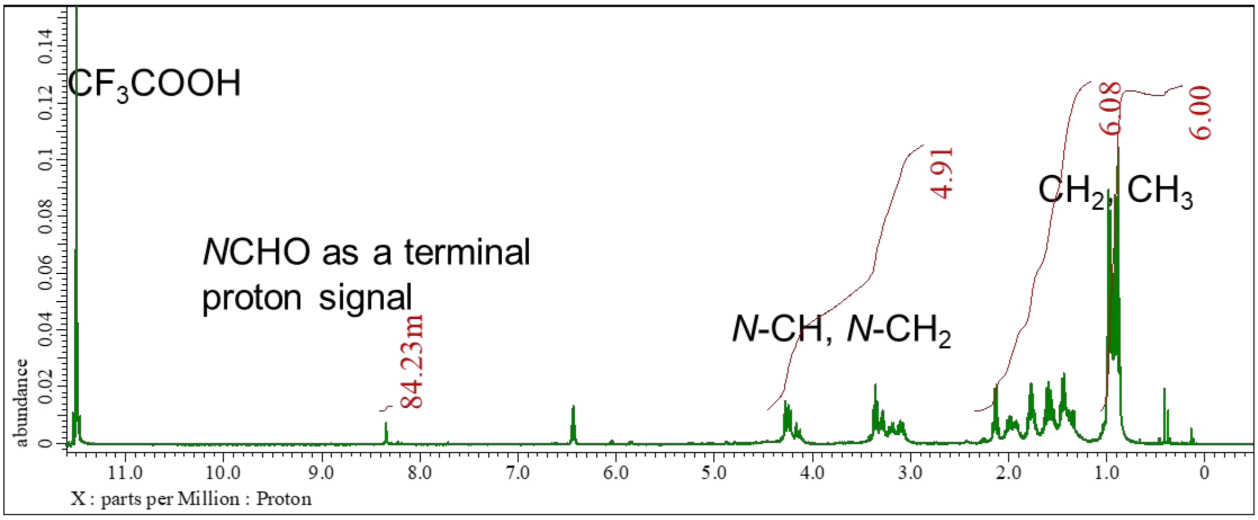

Figure S1. ^{1}H NMR spectrum of **Poly-C3-Pr** (400 MHz, CF_3COOD, 298 K).

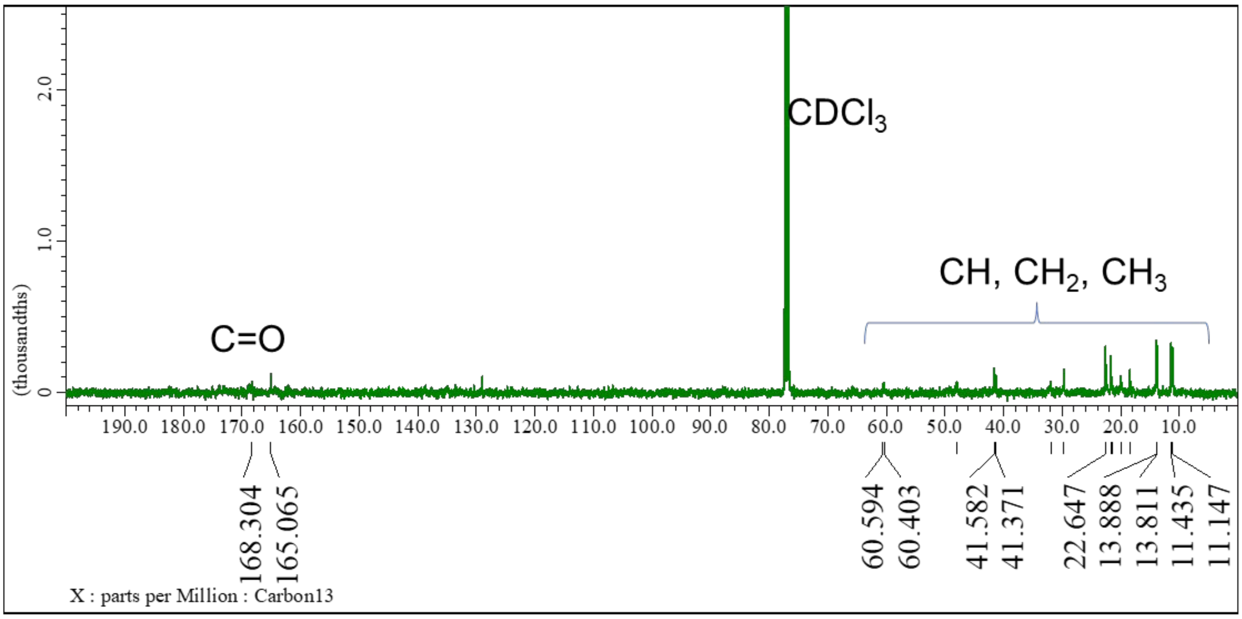

Figure S2. ^{13}C NMR spectrum of **Poly-C3-Pr** (100 MHz, $CDCl_3$, 298 K).

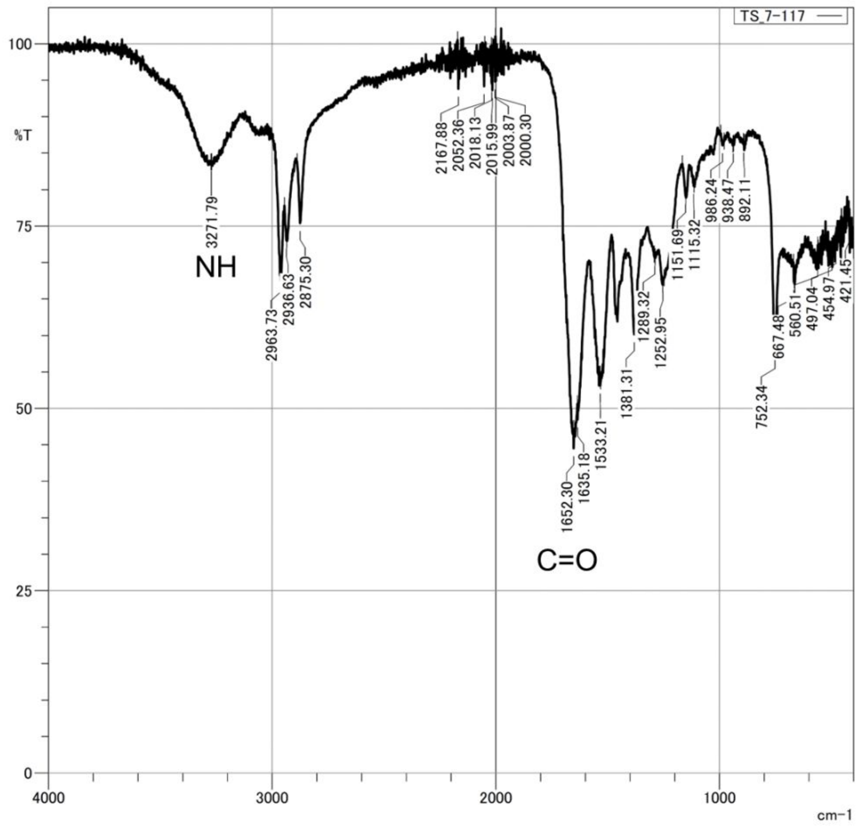

Figure S3. IR spectrum of **Poly-C3-Pr** (ATR).

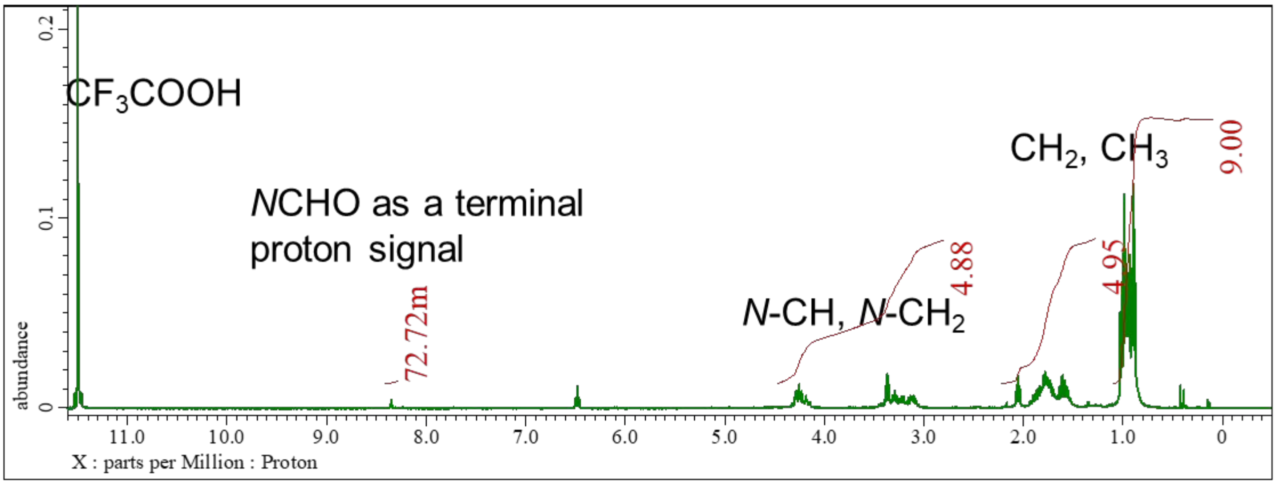

Figure S4. ^{1}H NMR spectrum of **Poly-iC4-Pr** (400 MHz, CF_3COOD, 298 K).

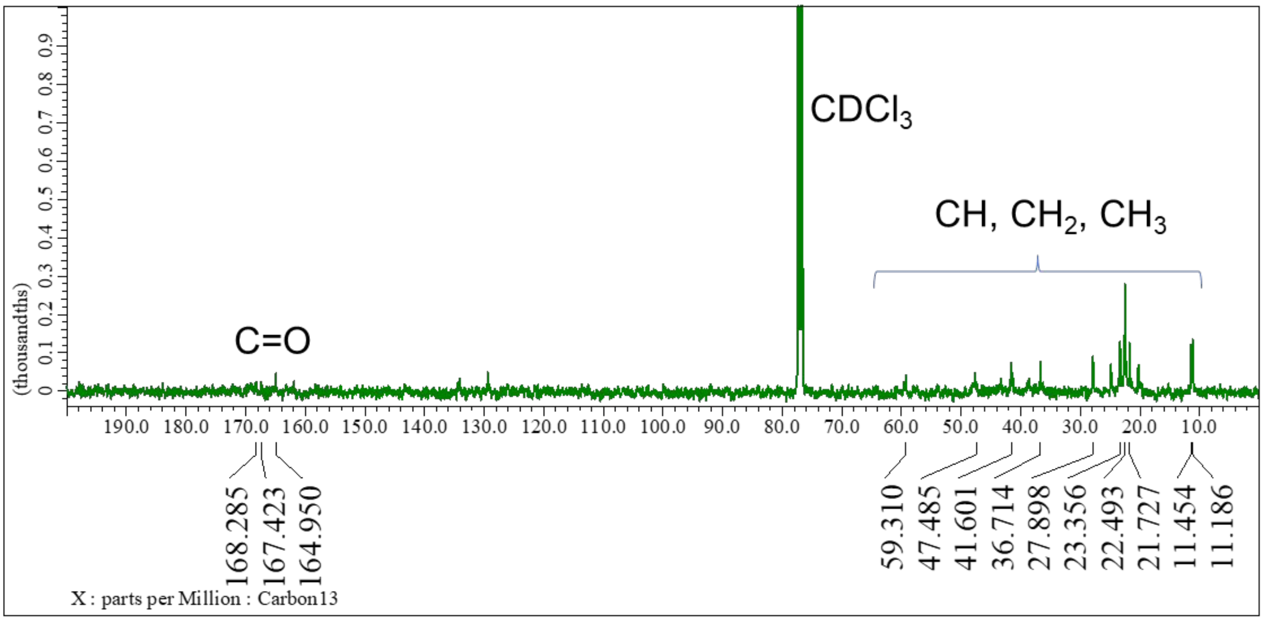

Figure S5. ^{13}C NMR spectrum of **Poly-iC4-Pr** (100 MHz, $CDCl_3$, 298 K).

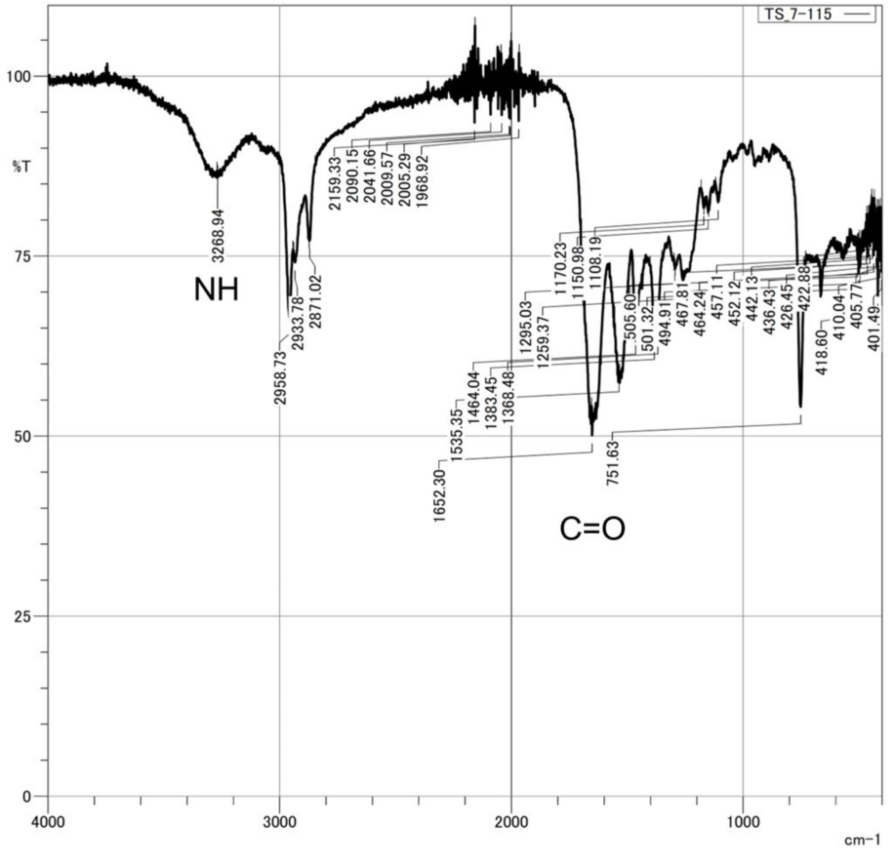

Figure S6. IR spectrum of **Poly-iC4-Pr** (ATR).

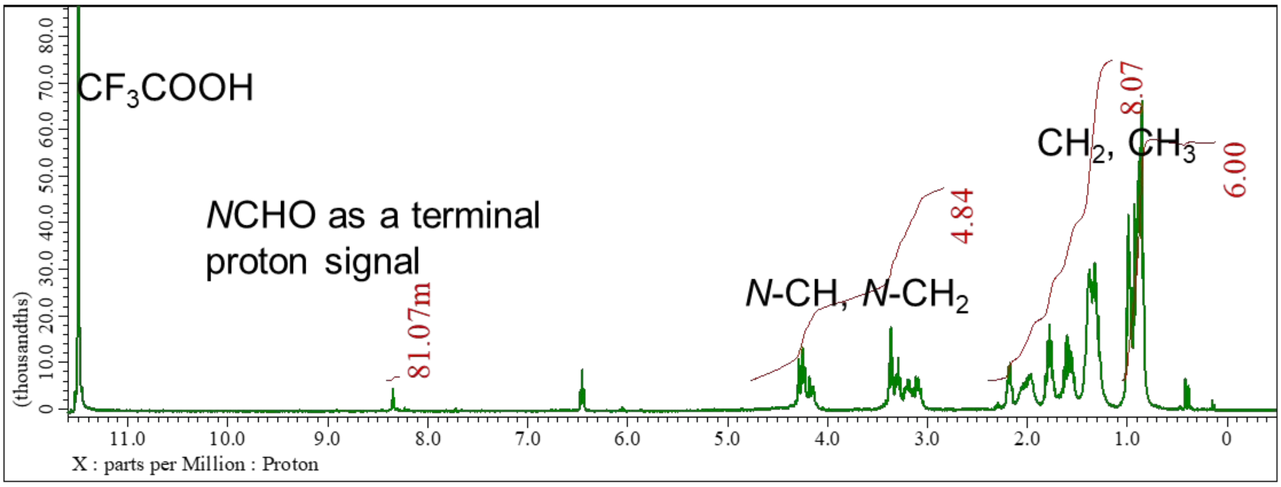

Figure S7. ^{1}H NMR spectrum of **Poly-C4-Pr** (400 MHz, CF_3COOD, 298 K).

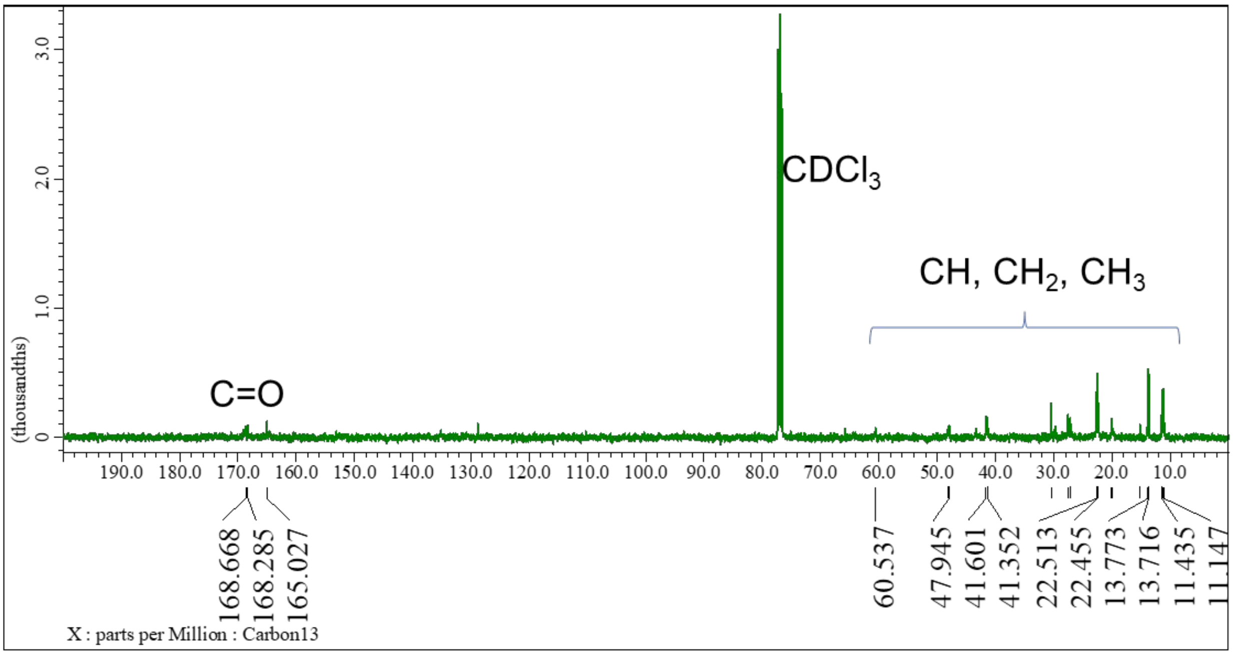

Figure S8. ^{13}C NMR spectrum of **Poly-C4-Pr** (100 MHz, $CDCl_3$, 298 K).

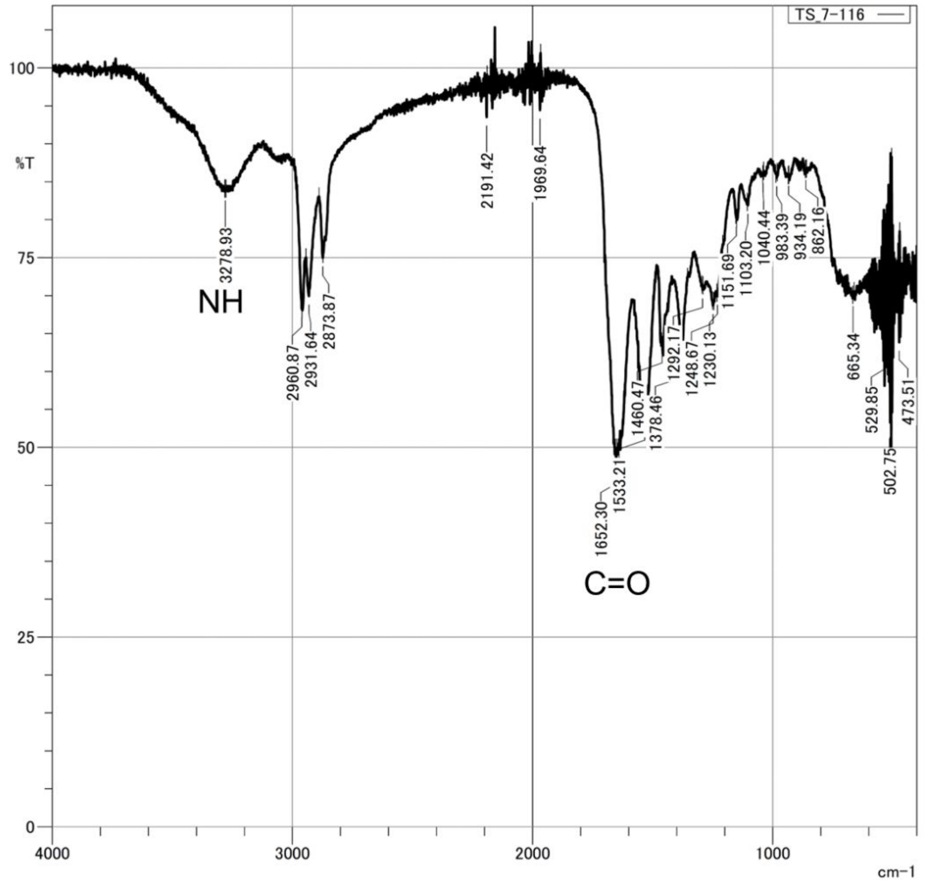

Figure S9. IR spectrum of **Poly-C4-Pr** (ATR).

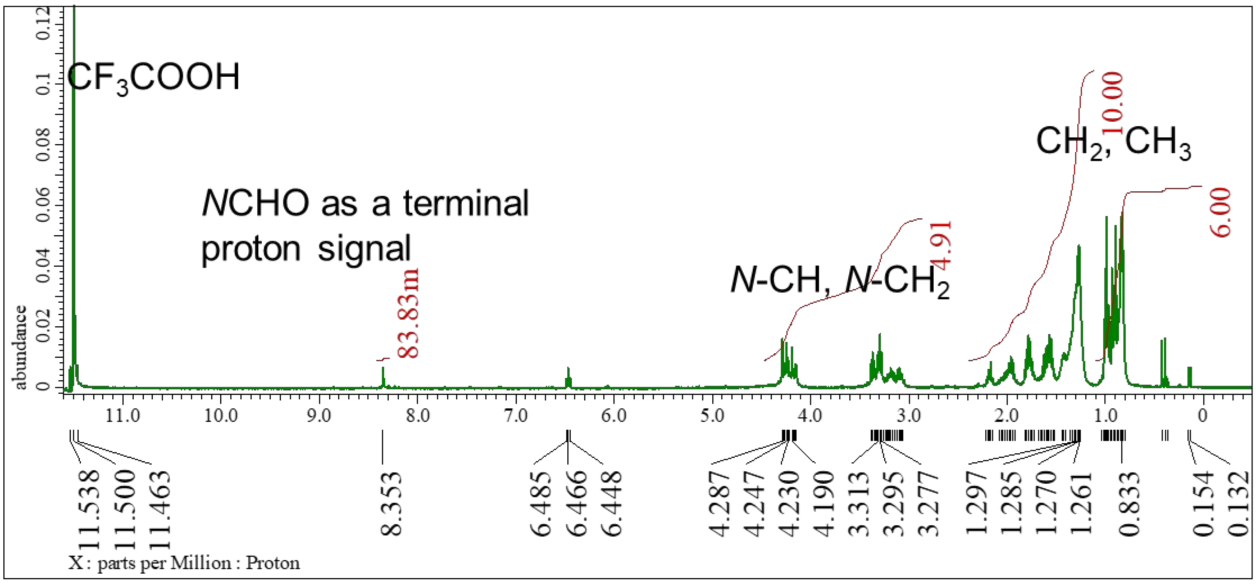

Figure S10. ^{1}H NMR spectrum of **Poly-C5-Pr** (400 MHz, CF_3COOD, 298 K).

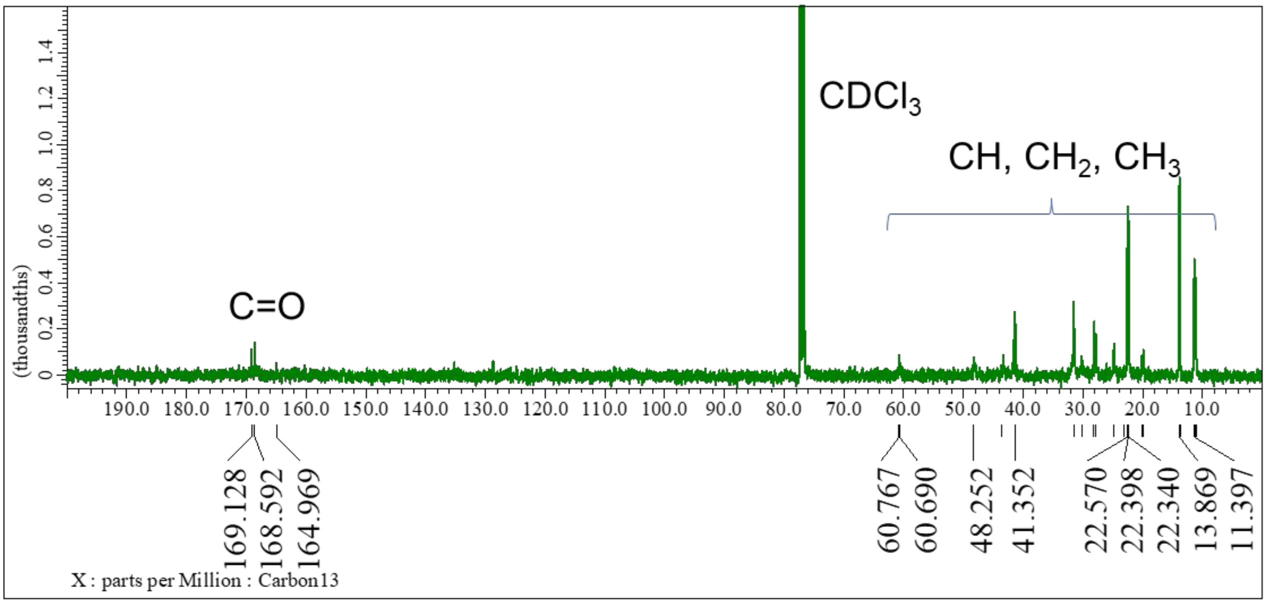

Figure S11. ^{13}C NMR spectrum of **Poly-C5-Pr** (100 MHz, $CDCl_3$, 298 K).

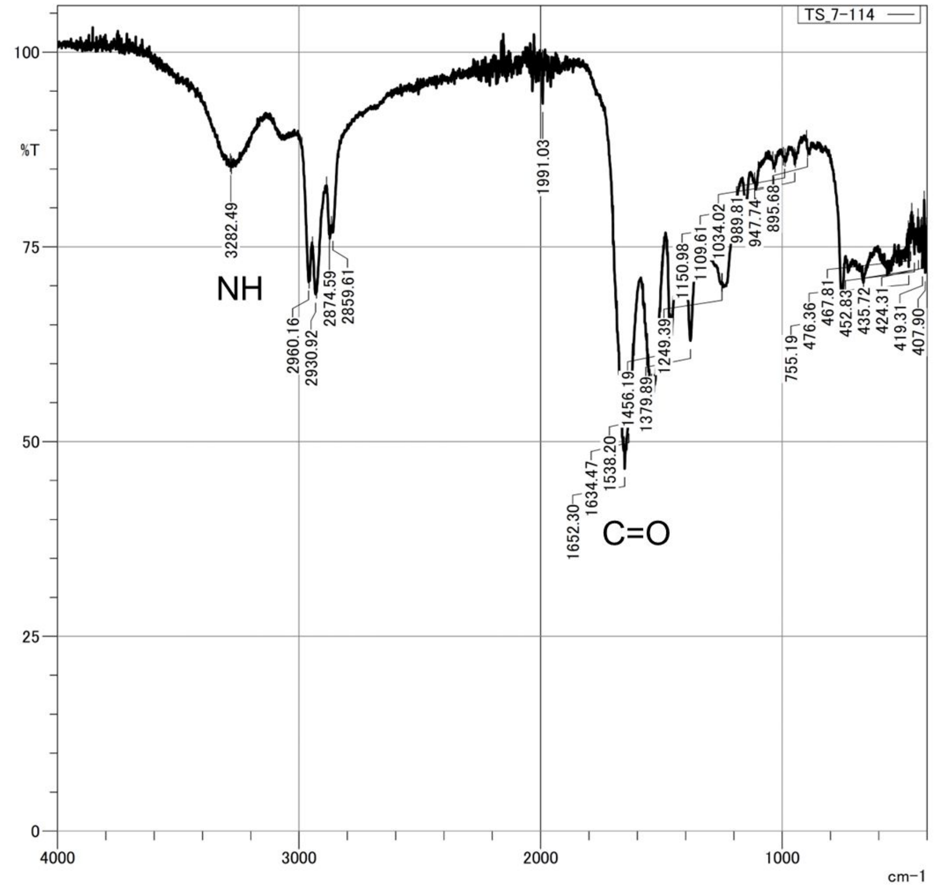

Figure S12. IR spectrum of **Poly-C5-Pr** (ATR).

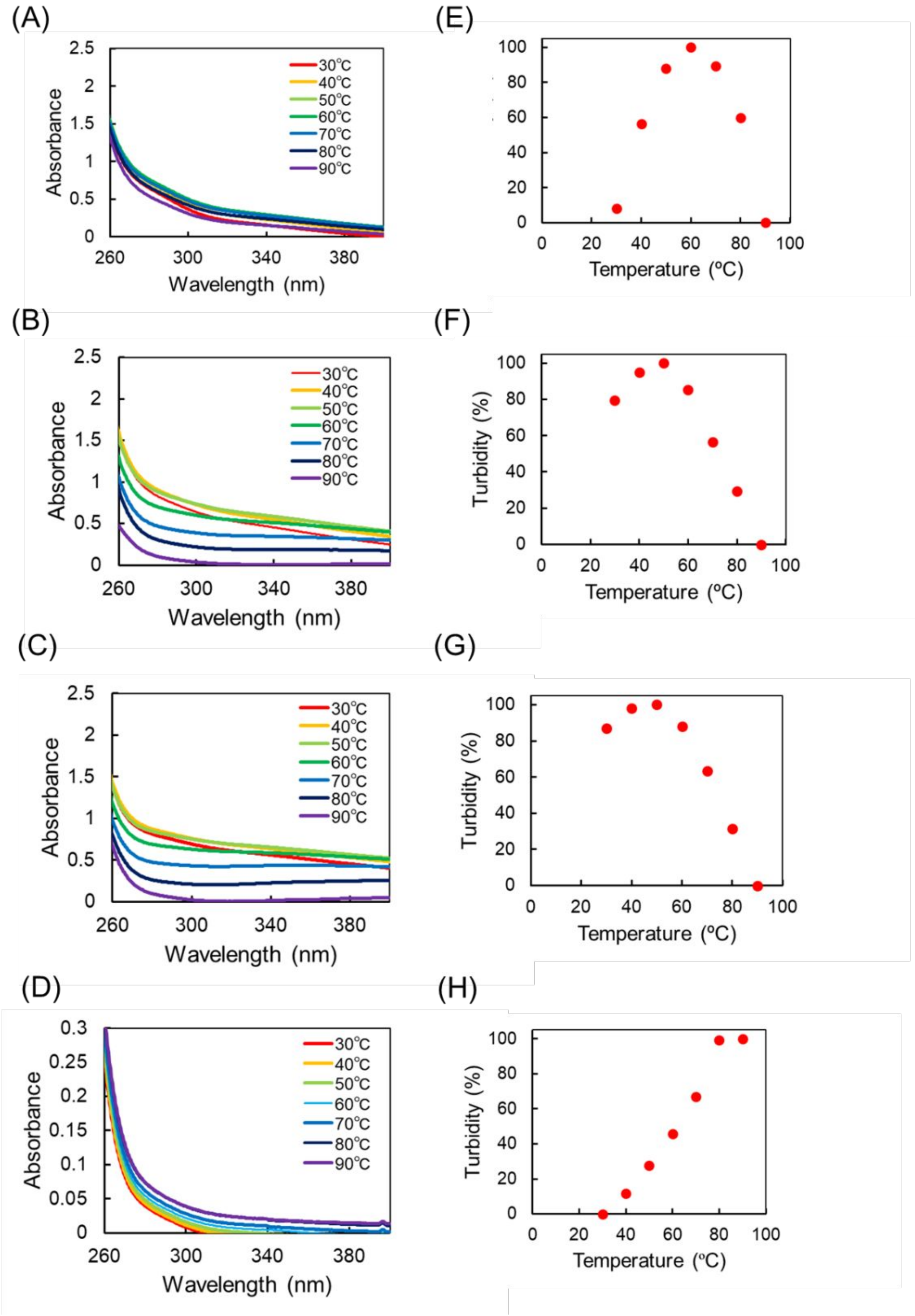

Figure S13. Temperature-dependency of UV-vis spectra of the polymer aqueous solutions upon heating: UV-vis spectra of (A) **Poly-C3-Pr**, (B) **Poly-iC4-Pr**, (C) **Poly-C4-Pr**, and (D) **Poly-C5-Pr** in aqueous media (0.5 wt%) at various temperatures and the temperature-dependence of transmittance of (E) **Poly-C3-Pr**, (F) **Poly-iC4-Pr**, (G) **Poly-C4-Pr**, and (H) **Poly-C5-Pr** at 330 nm as a function of concentration. The polymer sample (10.0 mg) was first dissolved in EtOH (100 μL) and then diluted with H_2O (1.9 mL) to give the corresponding aqueous solution. The UV-vis spectra at various temperatures were collected with 10 °C intervals upon heating from 30 to 90 °C. The waiting time for each temperature was 5 min.

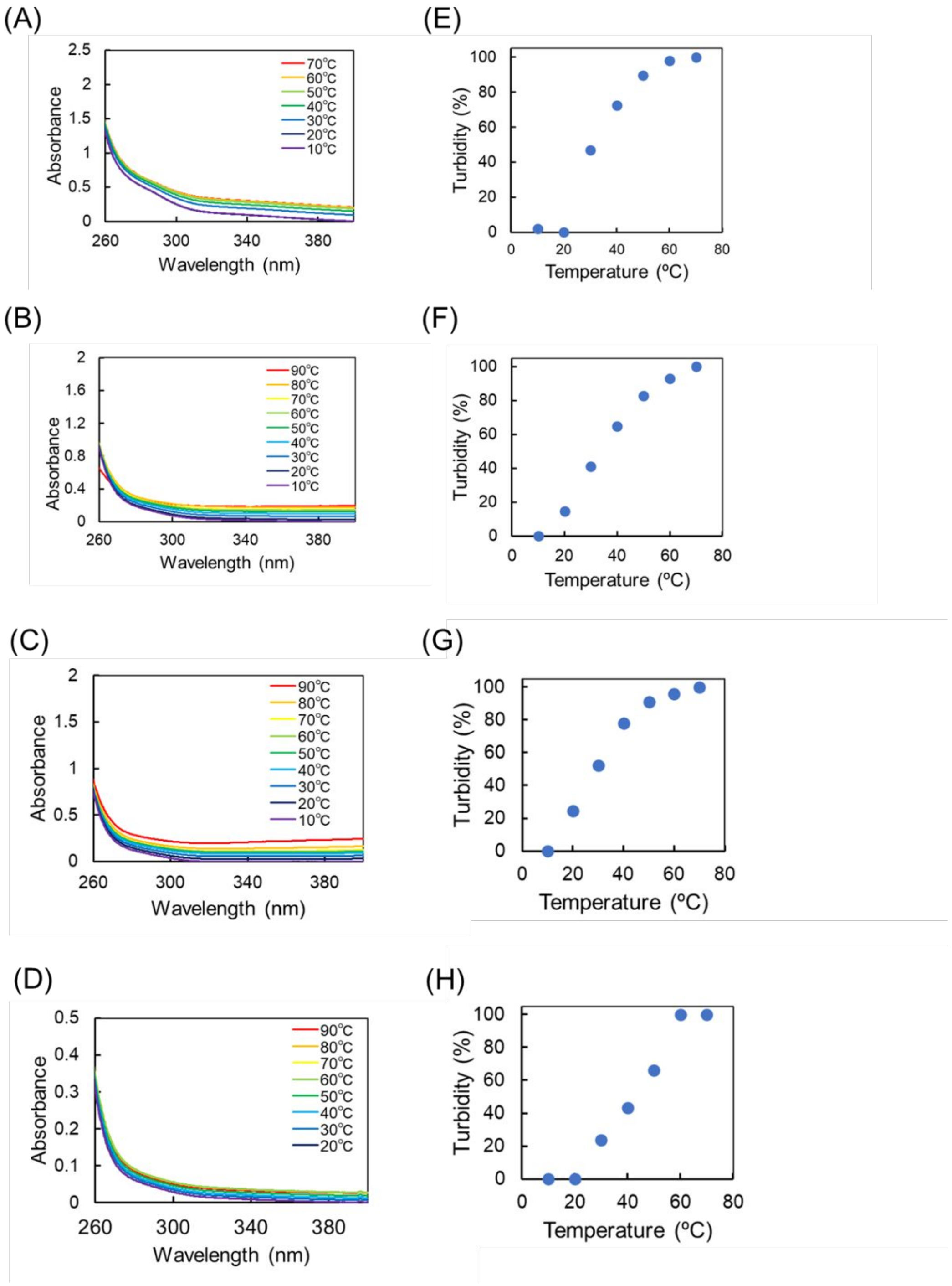

Figure S14. Temperature-dependency of UV-vis spectra of the polymer aqueous solutions upon cooling: UV-vis spectra of (A) **Poly-C3-Pr**, (B) **Poly-iC4-Pr**, (C) **Poly-C4-Pr**, and (D) **Poly-C5-Pr** in aqueous media (0.5 wt%) at various temperatures and the temperature-dependence of transmittance of (E) **Poly-C3-Pr**, (F) **Poly-iC4-Pr**, (G) **Poly-C4-Pr**, and (H) **Poly-C5-Pr** at 330 nm as a function of concentration. The polymer sample (10.0 mg) was first dissolved in EtOH (100 μL) and then diluted with H_2O (1.9 mL) to give the corresponding aqueous solution. The sample solutions were heated at 90 °C. Then, the UV-vis spectra at various temperatures were collected with 10 °C intervals upon cooling from 70 to 10 °C. The waiting time for each temperature was 5 min.
